# Utility of PIVKA-II and AFP in Differentiating Hepatocellular Carcinoma from Non-Malignant High-Risk Patients

**DOI:** 10.3390/medicina58081015

**Published:** 2022-07-28

**Authors:** Hana Hadi, Wan Muhammad Azfar Wan Shuaib, Raja Affendi Raja Ali, Hanita Othman

**Affiliations:** 1 Department of Pathology, Faculty of Medicine, Universiti Kebangsaan Malaysia, Hospital Canselor Tuanku Muhriz UKM, Jalan Yaacob Latif, Bandar Tun Razak, Cheras, Kuala Lumpur 56000, Malaysia; hanahadi5678@gmail.com (H.H.); drhanita@ppukm.ukm.edu.my (H.O.); 2 Gastroenterology Unit, Department of Medicine, Faculty of Medicine, Universiti Kebangsaan Malaysia, Hospital Canselor Tuanku Muhriz UKM, Jalan Yaacob Latif, Bandar Tun Razak, Cheras, Kuala Lumpur 56000, Malaysia; draffendi@ppukm.ukm.edu.my

**Keywords:** protein induced by vitamin K absence-II, alpha-fetoprotein, hepatocellular carcinoma, non-malignant high-risk group for HCC

## Abstract

*Background and Objectives:* We aim to compare the diagnostic performance of Protein induced by vitamin K absence-II (PIVKA-II), a biomarker for hepatocellular carcinoma (HCC), and alpha-fetoprotein (AFP) in differentiating HCC and non-malignant high-risk (NMHR) groups and to determine their cut-off values. *Materials and Methods:* A total of 163 patients, including 40 with HCC and 123 with NMHR (100 with liver cirrhosis and 23 with non-cirrhotic high-risk patients) were prospectively enrolled. The levels of AFP and PIVKA-II were measured, and their cut-off values were determined. We calculated and compared the areas under the receiver operating characteristic (AUROC) curves of PIVKA-II, AFP, and their combination. *Results:* The levels of PIVKA-II and AFP were found to be significantly higher in the HCC compared to NMHR patients (*p* < 0.0001). For the differentiation of HCC from NMHR, the optimal cutoff values for PIVKA-II and AFP were 36.7 mAU/mL (90% sensitivity; 82.1% specificity) and 14.2 ng/mL (75% sensitivity; 93.5% specificity), respectively. The AUROC of PIVKA-II (0.905, *p* < 0.0001) was higher compared to AFP (0.869, *p* < 0.0001), but the combination of PIVKA–II and AFP gave the highest AUROC value (0.911, *p* < 0.0001). However, their differences were not statistically significant (AFP vs. PIVKA; *p* = 0.4775, AFP vs. Combination; *p* = 0.3808, PIVKA vs. Combination; *p* = 0.2268). *Conclusions*: PIVKA-II and AFP showed equal performance in detecting HCC in high-risk patients. AFP as a screening marker for HCC may be adequate, and replacing or adding the PIVKA-II test in current clinical practice may be of little value.

## 1. Introduction

In 2020 it was reported that the sixth most diagnosed cancer is primary liver cancer which was also the third leading cause of cancer death around the world, accounting for 906,000 (4.7%) new cases and 830,000 (8.3%) deaths [1]. East Asia, particularly Mongolia and China, continue to lead in the overall burden of liver cancer, followed by the Southeast Asian region [1]. Among the various types, the most dominant form of primary cancer is Hepatocellular carcinoma (HCC) which accounts for 80% of all cases with diverse aetiologies [2]. The main risk factors for the development of HCC include chronic Hepatitis B virus (HBV) infection, Hepatitis C (HCV) infection, an aflatoxin-containing diet, as well as heavy alcohol consumption [3].

It is common practice to use serum AFP as a predictive biomarker for HCC and is generally associated with the size of the tumour [4]. Together with liver ultrasonography, it has increased the sensitivity for early-stage HCC detection [5]. Despite this, AFP also has limited sensitivity and specificity for early-stage HCC. For HCC tumours less than 5 cm in size, AFP has a sensitivity between 49% and 71% and a specificity between 49% and 86% [6]. It has also been reported that spurious elevations in AFP can be seen in active hepatic inflammation (e.g., viral hepatitis) or other liver masses such as cholangiocarcinoma [4]. Hence, the application of AFP alone plays a limited role in diagnosing early HCC.

The protein prothrombin is synthesized in the liver. The synthesis requires a vitamin K-dependent gamma-carboxylation process. However, patients with HCC have impaired gamma-carboxylation, which leads to the formation of Des-gamma-carboxyprothrombin, also known as a protein induced by vitamin K absence or antagonist-II (PIVKA-II). Liebman et al. reported in 1984 that PIVKA-II was significantly increased in the serum of HCC patients, and it could potentially be a marker for HCC [7]. Certain studies reported that PIVKA-II was superior to AFP and could potentially replace it to diagnose HCC [8]. Other studies have suggested that the combined detection of PIVKA-II and AFP may improve the HCC diagnosis compared to using each biomarker alone [9]. This raises the question of the diagnostic value of PIVKA-II and whether there is a correlation between PIVKA-II and AFP. Whether PIVKA-II can completely replace or supplement the role of AFP in the HCC diagnosis is still up for discussion [8,9].

To resolve these uncertainties and controversies, we aim to evaluate the diagnostic accuracy of PIVKA-II compared to AFP to differentiate HCC from non-malignant high-risk (NMHR) group patients at our centre and to determine their cut-off values. Many studies have been conducted in the East Asian region, particularly in China as well as in Europe. With some studies suggesting regional variability in the values of PIVKA-II and AFP, we are keen to investigate how this applies to the Southeast Asian region, particularly Malaysia, where minimal studies have been conducted.

## 2. Materials and Methods

### 2.1. Study Design & Patient Selection

A cross-sectional study was conducted over 12 months after approval by the research and ethics committee of Universiti Kebangsaan Malaysia Medical Centre (UKMMC). A total of 163 patients with HCC and those of high-risk groups in developing HCC who attended gastroenterology clinics or were admitted to gastroenterology wards, UKMMC, were prospectively enrolled. The patients were divided into HCC and non-malignant high-risk (NMHR) groups.

All patients with HCC were newly diagnosed and at the advanced stage. Samples were collected from patients before commencing any treatment modalities for HCC. The diagnosis of HCC was based on appropriate imaging characteristics as defined by accepted guidelines [10]. The tumour staging is defined according to the Barcelona clinic liver cancer (BCLC) staging classification [11]. Aetiolgy of the 40 HCC patients included 26 HBV, 12 NASH/NAFLD, and 2 HCV.

The NMHR group were patients affected by Chronic Hepatitis B Virus (HBV), Hepatitis C Virus (HCV), non-alcoholic steatohepatitis (NASH), and liver cirrhosis (LC). The diagnoses in this group were based on liver biochemistry, seropositivity for hepatitis B surface antigen (HBsAg) for a minimum of 6 months period, detection of antibodies against HCV (anti-HCV)/positivity for HCV RNA test, ultrasonographic/computerised tomography (CT) scan, or histopathology. The liver cirrhosis (LC) diagnosis was based on the clinical, laboratory, liver ultrasound, and fibro scan. Child–Pugh scores were calculated for liver cirrhosis and HCC patients. Excluded patients were those with alcoholic liver disease, warfarin consumption, recent vitamin K administration, and other malignancies.

### 2.2. Sample Size Calculation

The sample size was calculated based on specificity using the formula below.

Specificity of 96.8% was used based on a study by Seo SI et al. [12] and the prevalence of Hepatocellular Carcinoma of 4.9 per 100,000 population based on the Malaysian Cancer Statistics for liver cancer.

$$FP+TN=z^{2}\times \frac{\left(SP\left(1-SP\right)\right)}{W^{2}}$$

$$N\left(SP\right)=\frac{FP+TN}{\left(1-P\right)}$$

*FP* False Positive
*TN* True Negative
*Z* Value from the standard normal distribution reflecting the confidence level that will be used (e.g., *Z* = 1.96 for 95%)
*SP* Specificity
*W* Accuracy (0.05)
*N* Sample size (sample population)
*P* Prevalence

A sample size of N = 48 was calculated for each group. Since two main groups were being studied (Hepatocellular Carcinoma and Non-malignant High-Risk Group), a total sample size of 48 × 2 = 96 was required. However, for the Hepatocellular Carcinoma group, we only managed to attain 40 due to difficulty in finding patients. We managed to get 123 patients for the Non-malignant High-Risk Group (we included more even though 48 was sufficient to increase the power of the study). This gives us a total of 163 patients.

### 2.3. Laboratory Analysis

Blood samples were collected immediately after recruitment in the clinic or admission to the ward. A maximum of 4 mL of venous blood was collected in a plain tube and centrifuged immediately. The serum was aliquoted and stored frozen at −80 °C until analysis. Serum AFP levels were measured by a commercially available Chemiluminescent Microparticle Immunoassay (Architect, Abbott Diagnostic, Abbott Park, IL, USA). Serum PIVKA-II levels were measured by Chemiluminescent Microparticle Immunoassay (Architect, Abbott Diagnostic, Abbott Park, IL, USA) according to the manufacturer’s instructions.

### 2.4. Statistical Analysis

The categorical data were presented in frequencies (*n*) and percentages (%). Continuous data were presented in median and inter-quartile as the assumption of the data was not normally distributed based on the Kolmogorov–Smirnov/Shapiro–Wilk test. The Mann–Whitney U-test analysed a comparison between the median of the two groups for the non-parametric distribution of variables. For categorical variables, statistical differences between groups were assessed by the Chi-square test. Spearman correlation coefficients were used to determine the correlation between the tumour markers with laboratory parameters, Child–Pugh score, and tumour staging.

A new variable was used, the combination of AFP and PIVKA-II levels (log AFP + 4.6 * log PIVKA-II), conceived in a previous study [13]. A Receiver Operating Characteristic (ROC) curve was plotted. The areas under the receiver operating characteristic (AUROC) were measured and compared to evaluate the accuracy of AFP, PIVKA-II, and the combination. The best cut-off value was calculated, and the specificity and sensitivity of the parameters were determined using the best cut-off value. As an index of sensitivity and specificity, Youden’s index was calculated. In all statistical analyses, *p* < 0.05 (95% confidence interval) was considered significant. All clinical and laboratory data were stored and analysed using the Statistical Package for Social Sciences (SPSS) statistical software version 25 and Medcalc, version 18.

## 3. Results

### 3.1. Comparison of the Clinical Features and Baseline Characteristics between the HCC and NMHR Groups

A total of 163 patients (the majority are males) were recruited, and they were divided into three subgroups based on their diagnosis: (1) 40 with HCC; (2) 100 with liver cirrhosis without HCC; and (3) 23 with non-cirrhotic high-risk (NCHR) which included 12 patients with Chronic Hepatitis B, 4 with Hepatitis C, and 7 with non-alcoholic steatohepatitis.

All patients in NCHR (100%) and most liver cirrhosis (LC) patients (76%) had preserved liver function (Child-Pugh A). Patients in the HCC group had worse Child–Pugh scores, whereas most had Child–Pugh scores of B (40%) and C (32.5%).

Patients in the HCC group showed more advanced liver dysfunction than patients in the non-HCC group (LC and NCHR). The total bilirubin, alanine aminotransferase (ALT), and international normalized ratio (INR) levels were significantly higher in HCC patients than those without HCC, whereas albumin levels were significantly higher in patients without HCC. Table 1 summarises the baseline characteristics of these patients.

The values of PIVKA-II and AFP for different groups are listed in Table 2. The median levels (interquartile range) of both PIVKA-II and AFP were significantly higher in the HCC group compared to the LC group and the NCHR group [1170.1 (29,863) mAU/mL vs. 26.5 (13.9) mAU/mL vs. 26.4 (6.8) mAU/mL for PIVKA-II, 65.5 (1986.7) ng/mL vs. 2.8 (3.7) ng/mL vs. 3.1 (2.2) ng/mL for AFP, all *p* < 0.001].

Analysis of the correlation between tumour markers and blood parameters in all groups demonstrated that the severity of BCLC staging, Child–Pugh score, total bilirubin, ALT, and INR levels were positively correlated with both PIVKA-II and AFP levels (Table 3). There was a negative correlation between both markers with albumin levels.

### 3.2. Diagnostic Values for PIVKA-II and AFP in the Differentiation of HCC from NMHR

**Figure 1:**
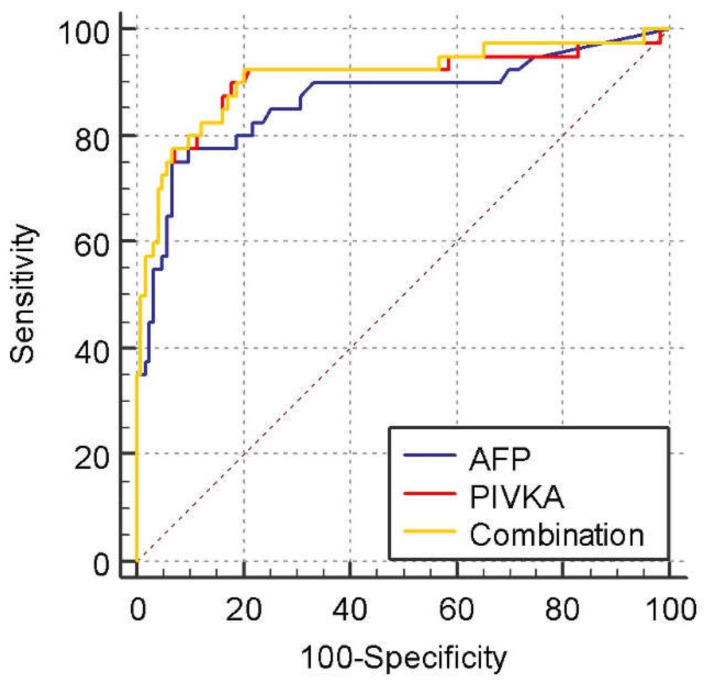
Receiver operating characteristic curves comparing PIVKA, AFP, and their combination in patients with HCC vs. those in the non-malignant high-risk group (NMHR). The area under the receiver operating characteristic curve was 0.905, 95% confidence interval [CI], 0.849 to 0.945, *p* < 0.0001 for PIVKA, 0.869, 95% CI, 0.807 to 0.916, *p* < 0.0001 for AFP, and 0.911, 95% CI, 0.856 to 0.950, *p* < 0.0001 for the combined AFP and PIVKA. *p* = 0.4775 for AFP vs. PIVKA-II, *p* = 0.2268 for Combination vs. PIVKA-II, and *p* = 0.3808 for Combination vs. AFP.

The ROC curves for tumor markers for diagnosing HCC from NMHR are shown in Figure 1. The AUROC curve of PIVKA-II (0.905, 95% confidence interval [CI], 0.849 to 0.945, *p* < 0.0001) was higher than that of AFP (0.869, 95% CI, 0.807 to 0.916, *p* < 0.0001), but their combination gave the highest AUROC value (0.911, 95% CI, 0.856 to 0.950, *p* < 0.0001) in differentiating HCC from NMHR. However, their differences were not statistically significant (AFP vs. PIVKA-II; *p* = 0.4775, AFP vs. Combination; *p* = 0.3808, PIVKA-II vs. Combination; *p* = 0.2268).

**Figure 2:**
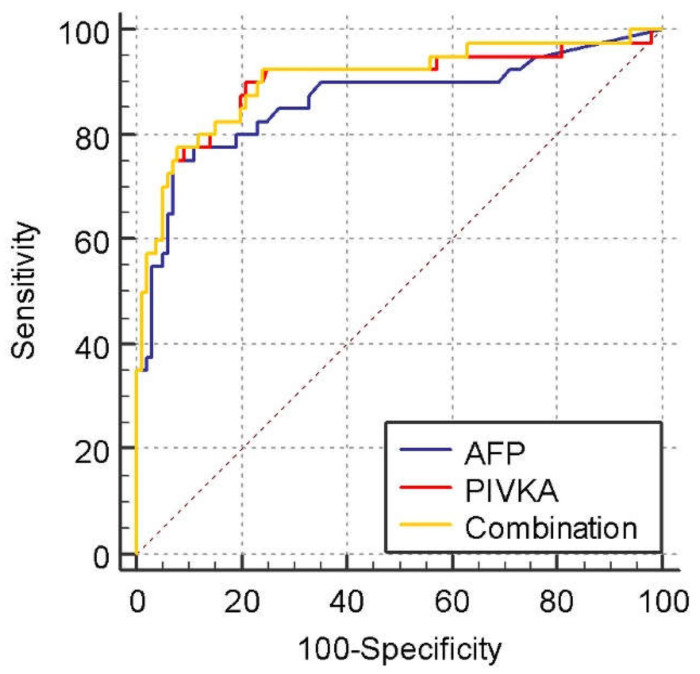
Receiver operating characteristic curves compare PIVKA, AFP, and both in patients with HCC vs. those with liver cirrhosis (LC). The area under the receiver operating characteristic curve was 0.898, 95% CI, 0.835 to 0.943, *p* < 0.0001 for PIVKA, 0.865, 95% CI, 0.797 to 0.917, *p* < 0.0001 for AFP, and 0.904, 95% CI, 0.842 to 0.947, *p* < 0.0001 for the combined AFP and PIVKA. *p* = 0.5121 for AFP vs. PIVKA-II, *p* = 0.2538 for Combination vs. PIVKA-II, and *p* = 0.4130 for Combination vs. AFP.

Figure 2 shows the ROC curves for tumor markers for diagnosing HCC from LC. The AUROC curve of PIVKA-II, AFP, and the combination were 0.898 (95% CI, 0.835 to 0.943, *p* < 0.0001), 0.865 (95% CI, 0.797 to 0.917, *p* < 0.0001), and 0.904 (95% CI, 0.842 to 0.947, *p* < 0.0001), respectively. Again, the differences were not statistically significant (AFP vs. PIVKA-II; *p* = 0.5121, AFP vs. Combination; *p* = 0.4130, PIVKA-II vs. Combination; *p* = 0.2538).

The best cut-off values in differentiating HCC and NMHR for PIVKA-II and AFP were 36.7 mAU/mL (Youden index J: 0.7211) and 14.2 ng/mL (Youden index J: 0.6850), respectively. The sensitivity and specificity at these cutoff values were 90% and 82.1%, respectively, for PIVKA-II and 75% and 93.5%, respectively, for AFP. Table 4 shows the sensitivity, specificity, positive predictive value (PPV), and negative predictive value (NPV) for different cut-off values differentiating HCC from NMHR and liver cirrhosis.

## 4. Discussion

HCC continues to exhibit a poor prognosis among those affected, especially with significant tumour burden and distant metastasis, but this is primarily attributed to the delay in presentation and initiation of treatment [14,15]. The treatment of hepatocellular carcinoma in patients without cirrhosis is hepatic resection, but few presents during this early stage [16].

Current practice for HCC surveillance includes biannual liver ultrasonography with or without serum alpha-fetoprotein (AFP) as recommended by the American Association for the Study of the Liver Diseases (AASLD), Asian Pacific Association for the Study of the Liver (APASL), and European Association for the Study of the Liver (EASL) [17,18]. Despite this, various discussions debated the variability of the findings, where a study found that male gender, body mass index, Child–Pugh class B or C cirrhosis, alcohol-related cirrhosis, NASH cirrhosis, and inpatient status as independent risk factors for inadequate ultrasound quality [19]. To rely solely on liver ultrasonography would yield low sensitivity for HCC surveillance hence the recommendation to have additional tests coupled with it.

In Malaysia, there have been recommendations to use AFP and/or liver ultrasonography to screen high-risk groups, specifically hepatitis B carriers (Asian males ≥ 40 years, Asian females ≥ 50 years), all cirrhotic hepatitis B regardless of age, family history of HCC, and liver cirrhosis (hepatitis C, alcoholic cirrhosis, genetic hemochromatosis, primary biliary cirrhosis), based on a report by the Malaysian Health Technology Assessment Section. However, this has yet to be considered a national cancer screening program as per the latest Malaysian National Strategic Plan for Cancer Control Programme (2021–2025).

We evaluated the usefulness of tumour markers in differentiating HCC in patients with cirrhosis and NCHR. AFP and PIVKA-II were analysed and compared when used alone or in combination. Our findings show that both markers were significantly higher in patients with HCC compared to NMHR patients (LC and NCHR). Both tumour markers also showed a positive correlation with the severity of BCLC staging, Child–Pugh score, total bilirubin, ALT and INR levels, and a negative correlation with albumin levels.

This study demonstrated that PIVKA-II and AFP are reliable in detecting HCC when separately used as a single marker. This contrasts with a study by Loglio et al., which concluded from their research that the combination of PIVKA-II and AFP increases the detection rate for HCC [20]. Our study did not show any statistically significant improvement in detecting HCC when combining the two markers compared to using a single marker. It is important to note that their study involved cirrhotic Caucasian patients and our patients were all South East Asians, specifically Malaysians. Demographics and racial background could probably play a role in the different outcomes attained. Furthermore, using a combination of two tumour markers is deemed not cost-effective.

Si et al. also demonstrated that the combination of AFP and PIVKA-II only slightly improved the diagnostic performance. The serum PIVKA-II level had a better diagnostic value than AFP. Here the study recruited Chinese patients to be evaluated, further strengthening the deduction that levels of tumour marker response may vary between races. The combination does not significantly diagnose HCC as per our findings [21].

Other studies have demonstrated that PIVKA-II is superior to AFP in detecting HCC [22,23]. Interestingly laboratory analysing methods and principles could also contribute to the different values attained. In some of the studies cited, enzyme-linked immunosorbent assays (ELISAs) were used to measure PIVKA-II levels and AFP was measured using the method of immunofluorescence on an automatic electrophoresis fluorescence immunoassay analyzer compared to our study where both analytes were measured using the chemiluminescent microparticle immunoassay method. Each method has its advantages and drawbacks which may lead to confounding results.

In the current study, PIVKA-II was found to have a statistically similar performance to AFP in detecting HCC. However, at the optimal cut-off values, the sensitivity of PIVKA-II (90%) is higher than AFP (75%), and this has been demonstrated by previous studies [12,24]. On the other hand, the specificity for AFP (93.5%) is higher than that of PIVKA-II (82.1%). The sensitivity is essential in screening because HCC surveillance aims to detect early HCC, and false-positive patients can be investigated with subsequent imaging. This finding may put PIVKA-II at an advantage over AFP from a screening point of view, even though we cannot deny that the results showed AFP has better specificity. Nevertheless, serum PIVKA-II may be falsely elevated in several conditions such as administration of warfarin, acute hepatic failure, and alcoholic liver disease [25]. The interpretation of PIVKA-II in these groups must be done with caution. Hence why we excluded patients with these conditions to avoid the confounding of results and interpretation.

The optimal cut-off value of PIVKA-II was 36.7 mAU/mL (90% sensitivity: 82.1% specificity) for the differentiation of HCC from NMHR. This is in line with a study by Parkash et al. where the optimum cut-off value in their study was 37.5 mAU/mL [26] but lower than the cut-off value of 50 mAU/mL by Su et al. [27] and much lower than the cut-off of 150 mAU/mL by Marrero et al. [28]. Interestingly, the 150 mAU/mL cut-off point was attained among patients whom the majority are early-stage HCC patients compared to our advanced-stage HCC patients. Here again, we postulate that the variable cut-off values in different populations are due to different racial and geographical backgrounds.

For AFP, the optimal cut-off value was 14.2 ng/mL (75% sensitivity; 93.5% specificity). The value is slightly higher than the study by Jasirwan et al. at 10 ng/mL [29] but significantly lower than the threshold of 400 ng/mL by Zhang et al. [30].

The cut-off values in differentiating HCC with NMHR and HCC with LC are probably similar because most subjects in NMHR comprise LC patients (Table 4). For AFP, raising the cut-off to 19 ng/mL and 196 ng/mL will reduce the sensitivity but increase the specificity. This is like PIVKA-II, where raising the cut-off to 100 mAU/mL will reduce the sensitivity and increase the specificity. This indicates more patients with false-negative results will be missed in HCC screening. However, increasing the specificity means that the patients who will be labelled negative are those who do not have the disease and will avoid unnecessary imaging and anxiety.

In this study, most control (NMHR) group patients were cirrhotic rather than chronic hepatitis patients or healthy people. This is because irrespective of its aetiology, liver cirrhosis is the most common cause of HCC, and cirrhotic patients are at the highest risk of developing HCC. It is more challenging to detect early HCC in the cirrhotic liver by ultrasound examination, which made us recruit more cirrhotic patients in the NMHR group.

However, it is important to note that some studies have also shown that AFP values differ between viral-associated HCC and NAFLD/NASH-associated HCC. Hepatitis B-associated HCC is reported to have higher values of AFP compared to other aetiologies. Li M et al. [31] concluded that higher levels of AFP among HBV-infected patients were due to the presence of HBV protein (HBx). HBx induces AFP receptor regulation causing an increase in AFP expression in HCC due to HBV infection. Yao M, et al. [32] and Zhang C et al. [33] claimed that HBV co-transcription factors could directly bind AFP gene promoters which would eventually increase its expression. These findings could explain why several studies from Hann et al. [34], Liu C et al. [35], and Murugavel KG et al. [36] reported higher AFP levels in HBV-associated HCC patients. Hepatitis C infection is also an important aetiology; however, elevated AFP levels are less frequently seen as reported by Kobeisy MA et al. [37] and Fattovich G et al. [38]. NAFLD/NASH-associated HCC is becoming increasingly common, particularly in western countries; however, the elevation of AFP levels associated with NAFLD/NASH or Fatty liver disease is believed to be not as significant as their viral counterparts. Interestingly a study by Best j et al. [39] revealed that AFP levels among NASH-associated HCC had median AFP values higher among German patients compared to Japanese patients at 16.8 ng/mL vs 6.25 ng/mL which further shows that the racial element is still an important factor.

There are a few limitations to this study. First of which is the number of patients with HCC (*n* = 40) is relatively small compared to the control group/NMHR (*n* = 123). However, this reflects the natural course of the disease. The 40 HCC patients that we acquired for the study had various aetiologies namely Hepatitis B infection, NAFLD/NASH, as well as Hepatitis C infection. As various studies have shown that AFP levels do differ based on the aetiology of the HCC, this could possibly create a potential bias in the results produced. This limitation again is due to the relatively small sample size of our HCC group. Our study also excluded the patients with recent administration of vitamin K, warfarin, acute hepatic failure, and alcoholic liver disease, and these conditions may falsely elevate the PIVKA-II level. These exclusions might result in an overestimation of PIVKA-II specificity. It is possible that its performance would be inferior during routine care when encountering the patients mentioned above. Overall, there was a low number of subjects enrolled, and further studies with a larger sample size are needed to obtain a more accurate result. A larger sample size particularly for the HCC group would allow us to delve further specifically into the aetiolgy of each HCC case.

## 5. Conclusions

In conclusion, PIVKA-II and AFP are reliable biomarkers for detecting HCC in high-risk patients. Both markers showed statistically similar performances in detecting HCC in high-risk patients. However, PIVKA-II was shown to have better sensitivity but less specificity than AFP at the optimal cut-off values chosen. PIVKA-II might have an added value in surveillance of HCC, and AFP might be more specific for advanced HCC; therefore, further research with a bigger sample size is warranted. For the time being, the utilisation of AFP as a screening marker for HCC may be adequate at least among the Malaysian population in our centre.

## Figures and Tables

**Table 1:** Baseline characteristics of the study population.

	Non-Malignant High-Risk Group		HCC	*p*-Value
	Non-Cirrhotic High-Risk Group	Liver Cirrhosis		
Number of patients (*n*)	23	100	40	
Age, median (IQR)	50 (17)	63 (14)	64.5 (12)	0.001 ^b^
Gender				
Male	17 (73.9%)	70 (70%)	34 (85%)	
Female	6 (26.1%)	30 (30%)	6 (15%)	
Child-Pugh score				<0.0001 ^a^
A	23 (100%)	76 (76%)	11 (27.5%)	
B	-	15 (15%)	16 (40%)	
C	-	9 (9%)	13 (32.5%)	
BCLC stage	NA	NA		
A			7 (17.5%)	
B			10 (25%)	
C			17 (42.5%)	
D			6 (15%)	
Albumin, median (IQR)	40 (4)	35(9)	27 (10)	<0.0001 ^b^
TB	13 (6)	20 (24)	32.5 (50.3)	<0.0001 ^b^
ALT	23 (25)	30 (26)	46 (60)	0.002 ^b^
INR	1.0 (0)	1.2 (0.2)	1.3 (0.3)	<0.0001 ^b^

Data expressed as the number (percentage) and median (IQR); ^a^ by Pearson chi-square, ^b^ by Kruskal Wallis, statistically significant at *p* < 0.05.

**Table 2:** Values of AFP and PIVKA in HCC, LC, and non-cirrhotic high-risk (NCHR) groups.

**Parameters**	**LC**	**HCC**	**Statistical Value**	***p*-Value**
AFP, ng/ml	2.8 (3.7)	65.5 (1986.7)	541.5 ^a^	*p* < 0.001 ^a^
PIVKA, mAU/ml	26.5 (13.9)	1170.1 (29863)	408.5 ^a^	*p* < 0.001 ^a^
**Parameters**	**NCHR**	**HCC**	**Statistical value**	***p*-Value**
AFP, ng/ml	3.1 (2.2)	65.5 (1986.7)	105 ^a^	*p* < 0.001 ^a^
PIVKA, mAU/ml	26.4 (6.8)	1170.1 (29863)	61 ^a^	*p* < 0.001 ^a^

Values are expressed as median (IQR) ^a^ by Mann–Whitney U test, with Statistical significance at *p* < 0.05.

**Table 3:** Correlation of serum AFP and PIVKA with blood parameters, Child–Pugh score, and tumour staging.

Parameters	AFP Value		PIVKA Value	
	Correlation Coefficient (r)	*p*	Correlation Coefficient (r)	*p*
Child-Pugh score	0.32	<0.0001 ^a^	0.45	<0.0001 ^a^
BCLC staging	0.46	0.003 ^a^	0.46	0.003 ^a^
Albumin	−0.31	<0.0001 ^a^	−0.38	<0.0001 ^a^
TB	0.29	<0.0001 ^a^	0.29	<0.0001 ^a^
ALT	0.39	<0.0001 ^a^	0.29	<0.0001 ^a^
INR	0.25	0.001 ^a^	0.26	0.001 ^a^

^a^ by Spearman Correlation coefficient. Correlation is significant at the 0.05 level (2-tailed).

**Table 4:** Sensitivity, specificity, PPV, and NPV for different cut-off values of tumour markers in distinguishing hepatocellular carcinoma from NMHR and liver cirrhosis.

Cut-Off Value	Sensitivity (%)	Specificity (%)	PPV (%)	NPV (%)
**AFP (ng/mL)**				
HCC vs. NMHR: >14	75	93.5	26.3	99.2
HCC vs. LC: >14	75	93	48.2	97.7
HCC vs. NMHR: >19	62.5	94.3	25.4	98.8
HCC vs. LC: >19	62.5	94	47.5	96.6
HCC vs. NMHR: >196	37.5	98.4	41.6	98.1
HCC vs. LC: >196	37.5	98	62	94.7
**PIVKA (mAU/mL)**				
HCC vs. NMHR: >37	90	82.1	13.5	99.6
HCC vs. LC: >37	90	79	27.1	98.9
HCC vs. NMHR: >100	77.5	92.7	24.7	99.3
HCC vs. LC: >100	77.5	91	42.8	97.9

## Data Availability

The data presented in this study are available on request from the corresponding author.

## Abbreviations

AASLD, American Association for the Study of the Liver Diseases; AFP, alpha-fetoprotein; ALT, alanine aminotransferase; APASL, Asian Pacific Association for the Study of the Liver; AUROC, areas under the receiver operating characteristic; BCLC, Barcelona clinic liver cancer; CT, computerized tomography; EASL, European Association for the Study of the Liver; HBsAg, hepatitis B surface antigen; HBV, hepatitis B virus; HCC, hepatocellular carcinoma; HCV, hepatitis C virus; HCV RNA, hepatitis C virus ribonucleic acid; INR, international normalized ratio; LC, liver cirrhosis, NASH, non-alcoholic steatohepatitis; NCHR, non-cirrhotic high risk; NMHR, non-malignant high risk; PIVKA-II, protein induced by vitamin K absence; ROC, receiver operating characteristics; UKMMC, Universiti Kebangsaan Malaysia Medical Centre.
